# Description of a Non-Canonical AsPt Blue Species Originating from the Aerobic Oxidation of AP-1 in Aqueous Solution

**DOI:** 10.3390/ijms25137408

**Published:** 2024-07-05

**Authors:** Damiano Cirri, Tiziano Marzo, Piero Mastrorilli, Valentina Petrelli, Stefano Todisco, Elvira De Giglio, Cristina Gellini, Marilena Ricci, Alessandro Pratesi, Luigi Messori

**Affiliations:** 1 Department of Chemistry and Industrial Chemistry, University of Pisa, Via G. Moruzzi 13, 56124 Pisa, Italy; damiano.cirri@unipi.it; 2 Department of Pharmacy, University of Pisa, Via Bonanno Pisano 6, 56126 Pisa, Italy; tiziano.marzo@unipi.it; 3 Department of Civil, Environmental, Land, Building and Chemical Engineering (DICATECh), Polytechnic University of Bari, Via Orabona 4, 70125 Bari, Italy; pietro.mastrorilli@poliba.it (P.M.); valentina.petrelli@poliba.it (V.P.); stefano.todisco@poliba.it (S.T.); 4 Department of Chemistry, University of Bari “Aldo Moro”, Via Orabona 4, 70125 Bari, Italy; elvira.degiglio@uniba.it; 5 Department of Chemistry “Ugo Schiff”, University of Florence, Via della Lastruccia 3-13, 50019 Sesto Fiorentino, Italy; cristina.gellini@unifi.it (C.G.); marilena.ricci@unifi.it (M.R.)

**Keywords:** platinum blue compounds, arsenoplatin-1, CP/MAS ^1^H-^13^C, XPS

## Abstract

The peculiar behavior of arsenoplatin-1, ([Pt(µ-NHC(CH_3_)O)_2_ClAs(OH)_2_], AP-1), in aqueous solution and the progressive appearance of a characteristic and intense blue color led us to carry out a more extensive investigation to determine the nature of this elusive chemical species, which we named “AsPt blue”. A multi-technique approach was therefore implemented to describe the processes involved in the formation of AsPt blue, and some characteristic features of this intriguing species were revealed.

## 1. Introduction

**Figure 1:**
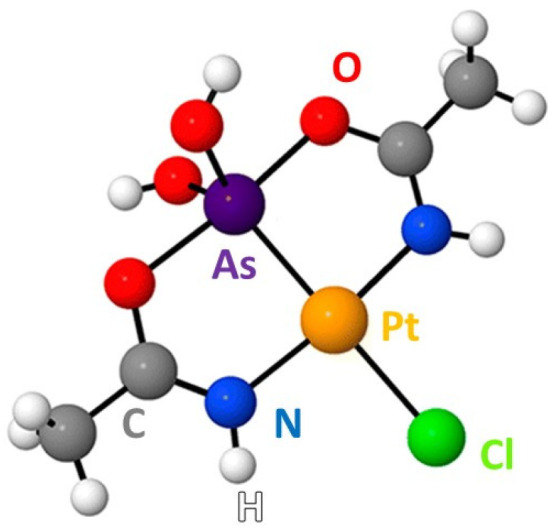
Chemical structure of AP-1. Reproduced and adapted with permission from reference [2].

Arsenoplatin AP-1 ([Pt(µ-NHC(CH_3_)O)_2_ClAs(OH)_2_]) is a promising dual-function inorganic drug that was developed and characterized in the laboratory of Thomas O’Halloran a few years ago as a prospective anticancer agent [1,2]. The chemical structure of AP-1 is shown in Figure 1. AP-1 is a chimeric species possessing an arsenous acid moiety bound to a platinum(II) center with an uncommon five-coordinate As(III) geometry.

The presence of the Pt-coordinated As(III) center makes the reactivity of AP-1 markedly different from that of cisplatin. The replacement of the chloride ligand with other small ligands in aqueous solutions is rapid at room temperature because of the very strong *trans* effect of the arsenic atom [1].

Notably, this chimeric complex, arising from the condensation of a cisplatin-like Pt(II) moiety with the arsenite anion, combines into a single molecular entity the favorable pharmacological properties of both cisplatin and trisenox, two FDA-approved inorganic anticancer drugs. The activation of AP-1 seems to rely on the cleavage of the Pt-As bond. Evidence for the progressive slow breaking of this bond within the cellular milieu was recently gained [2]. Further mechanistic, biological, and pharmacological studies on AP-1 are in progress [3].

Remarkably, when working with AP-1 aqueous solutions in the pH range of 5.5–8, we noticed that an intense blue color slowly develops with time under a variety of experimental conditions; the blue color is attributed to the progressive formation of a novel Pt-containing species (referred as AsPt blue hereafter).

The first platinum “blue” compound was prepared by the German chemists Hofmann and Bugge at the beginning of the 20th century [4]. Notably, after more than one hundred years since the first discovery of these derivatives, several examples of platinum “blues” have been reported in the literature. To the best of our knowledge, these elusive compounds are related to polynuclear Pt-Pt structures, in which an unusual Pt(III) center is present in the metal bond chain, typically containing four Pt atoms (with an average Pt oxidation number of 2.25) [5,6,7]. Moreover, a few reported papers have also described the existence of mononuclear Pt(III) blue compounds, in which the presence of bulky ligands seems to hamper the formation of a Pt-Pt direct bond [8,9]. In this frame, the aim of the present study is to obtain insights into the molecular speciation of AP-1 in the above-mentioned conditions and possibly gain information about the formed blue compound. It would be of great interest to discriminate between the mononuclear and polynuclear natures of the formed blue species and investigate the presence of arsenic atoms in the chromophore.

## 2. Results and Discussion

### 2.1. Formation of AsPt Blue in Solution

**Figure 2:**
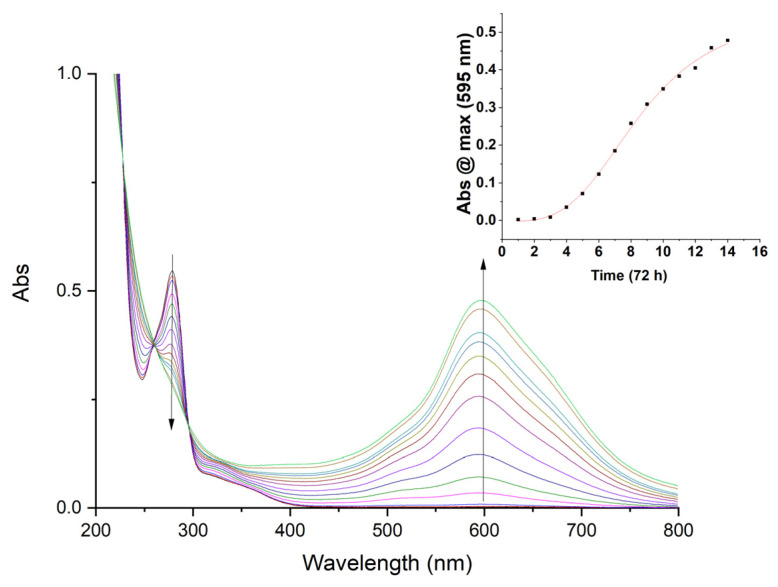
Time-dependent spectrophotometric profiles showing Pt blue formation. AP-1 (5 × 10^−4^ M) incubated for up to 72 h in the presence of 10 mM phosphate buffer, pH = 7.4. The inset plot shows the variation in absorbance monitored at 595 nm. Fourteen time points from t = 0 to t = 72 h were considered.

In our case, the kinetics of the process of AsPt blue formation is greatly influenced by the applied solution conditions. Indeed, through a series of explorative experiments, we were able to establish that the formation of Pt blue is strongly favored by the presence of phosphate or carbonate ions in the solution and inhibited by an excess of chloride ions or by an acidic environment (pH < 5). Even more importantly, we observed that no blue color develops when working with the exclusion of dioxygen; this latter observation strongly suggests that AsPt blue is indeed an oxidation product of AP-1. A typical spectrophotometric profile documenting the formation of AsPt blue in 10 mM phosphate buffer at pH 7.4 is depicted in Figure 2, where the lowering of the LMCT band at 278 nm (ε = 1092 L mol^−1^ cm^−1^), previously attributed to AP-1 [1], is clearly visible due to its hydrolysis. At the same time, an intense and broad absorption band progressively grows around 595 nm, with two evident shoulders at 520 and 670 nm [10,11].

The evolution of the blue band was monitored for 72 h. The formation of the blue species is relatively slow, follows a typical sigmoidal kinetic profile, and is still incomplete after 72 h of observation, although it is approaching saturation (Figure 2). Interestingly, the characteristic shape of the absorbance increase at 595 nm versus time suggests the existence of an initial induction period before the process of AsPt blue formation can begin (see Supporting Materials Table S1 for fitting parameters). This evidence is consistent with an autocatalytic process where the system should undergo a pre-activation step before starting to produce the blue species. This is something that is typical of several catalytic reactions, where the precatalyst converts into the actual catalytic species that determines the sigmoid shape of the reaction itself [12]. In this context, we can speculate that the induction period is due to the mechanism underlying the formation of the blue species itself. Indeed, the latter should form with the concomitant release of the chloride ligand from AP-1 and the coordinative assistance of the phosphate anion, together with the oxidizing atmospheric dioxygen. This hypothesis is well supported by further evidence that the formation of AsPt blue is inhibited by an acidic environment, the addition of NaCl, or working in an inert atmosphere. 

The formation of the AsPt blue species was then analyzed using ^1^H NMR measurements. Time-dependent ^1^H NMR spectra of freshly prepared solutions of AP-1 showed progressive broadening of the ^1^H signals, a behavior that is tentatively ascribed to AP-1 oligomerization. To support this hypothesis, the Pt blue species was passed through filters of increasing cutoffs (Figure S1). We observed that the freshly prepared AsPt blue species was able to cross filters with cutoffs of 3 kDa. Regardless, in aged solutions, the AsPt blue chromophore could not cross filters with cutoffs of 3 kDa nor 10 kDa. This observation supports the view that the formation of the AsPt blue species encompasses both oxidation and oligomerization processes; apparently, the molecular masses of the resulting oligomers grow to above 10 kDa. As stated above, in the absence of dioxygen, no AsPt blue formation is observed, yet a similar AP-1 oligomerization process takes place, leading to the formation of a scarcely soluble Pt(II) species that we earlier called Pt white; this species was preliminarily characterized in a previous work of ours [13].

### 2.2. Pt White: A More Advanced Characterization

**Figure 3:**
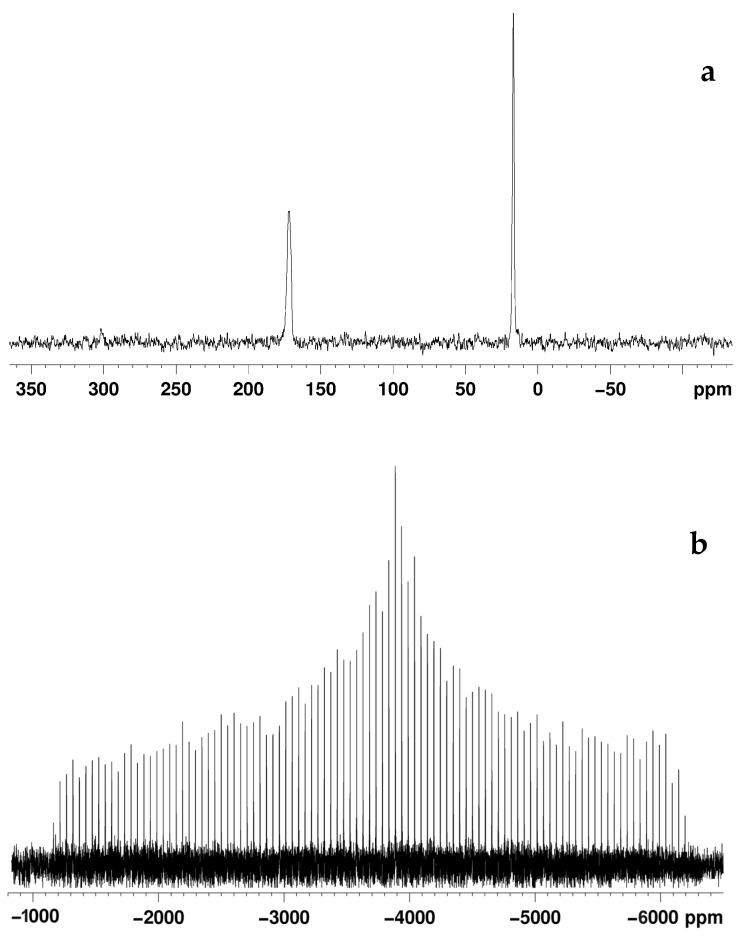
(**a**) CP/MAS ^1^H-^13^C and (**b**) static ^1^H-^195^Pt CP/CPMG spectra of Pt white.

The Pt white species has been further characterized here, as it can serve as a valuable basis of comparison for AsPt blue. The CP/MAS ^1^H-^13^C spectrum of Pt white (Figure 3a) shows two signals at δ 16.9 and δ 171.7, ascribable to CH_3_ and CONH of Pt-coordinated acetamidate ligands. The corresponding solid-state static ^195^Pt NMR spectrum is shown in Figure 3b and reveals that the isotropic ^195^Pt chemical shift is δ_iso_ = −3746 ppm. The values for the chemical shifts of the three tensors are δ_11_ = −1158 ppm; δ_22_ = −3885 ppm; δ_33_ = −6196 ppm. Such values are comparable to those found for AP-1 (whose solid-state static ^195^Pt NMR spectrum is reported in the SI) for which δ_iso_ = −3643 ppm; δ_11_ = −1000 ppm; δ_22_ = −3734 ppm; δ_33_ = −6196 ppm, indicating that the structure of the two species is roughly similar.

**Scheme 1:**
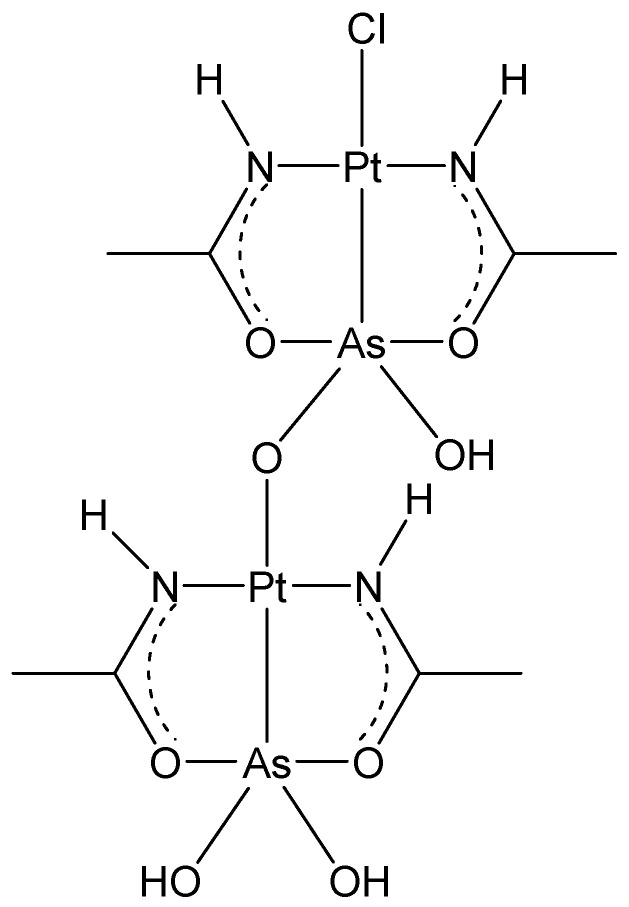
Initial step of Pt white formation.

We demonstrated that Pt white originated from the polymerization of AP-1, wherein a hydroxide of another AP-1 moiety replaces the chloride ligand (see Scheme 1). Interestingly, this attribution is in nice agreement with our previous results concerning the proposed crystallographic model of the Pt white oligomeric species [13]. According to this model, the Pt white species features a dendritic growth process—with chloride ligand release—through the interaction of the two hydroxyl oxygens of an arsenous moiety of an AP-1 molecule with Pt atoms belonging to different AP-1 molecules.

### 2.3. AsPt Blue: Preparation, Analytical Results, and Absorption Spectra

The above observations concerning the likely process of Pt blue formation prompted us to carry out further and more accurate studies to prepare, isolate, and characterize this novel Pt blue species in larger amounts. Based on several trials, a preparative procedure was determined to maximize the formation of this product. The procedure consists of dissolving AP-1 in 50 mM phosphate buffer at a pH of 7.4 in the presence of oxygen under continuous stirring. The blue color fully develops within 72 h. The AsPt blue species is then recovered through lyophilization of the aqueous buffer, shaking in methanol of the residual solid, and filtration of the formed suspension. The deep blue solution is then evaporated on a gently warmed watch glass, and the AsPt blue species is recovered by scratching the thin black layer formed on the glass. Extensive analytical data were collected for the AsPt blue samples, which were compared with those of AP-1 and Pt white (see Table 1). In the elemental composition of AsPt blue, the total Pt + As content is significantly reduced compared to AP-1 and Pt white, and the As content experiences a far larger decrease than Pt; an increase in the oxygen percentage is noted as well. Chlorine is retained at a percentage similar to that of AP-1. These results suggest that the oxidation process probably involves an appreciable loss of platinum and a more extensive loss of arsenic, implying partial cleavage of the As-Pt bond. It is worth noting that these data were obtained through the combination of different analytical techniques, i.e., elemental analysis (for C, H, N, O, and Cl) and ICP-OES (for Pt and As). Moreover, the determination of the elemental composition becomes less and less accurate as the molecular size increases, and, realistically, AsPt blue is a large oligomeric species [14]. Considering all the above, we determined the deviation obtained from the theoretical 100% value to be acceptable and in agreement with the errors deriving from the use of different techniques.

The electronic absorption spectrum was recorded for the Pt blue species dissolved in phosphate buffer at a pH of 7.4. The spectrum closely corresponds to the spectrum reported in Figure 2, with a broad and intense band centered at 600 nm. Attempts were also made to record an EPR spectrum for the AsPt blue samples in solution: the virtual lack of EPR signals points out that AsPt blue is predominantly diamagnetic in nature, this being nicely consistent with the NMR results (see below).

### 2.4. AsPt Blue: NMR Studies

**Figure 4:**
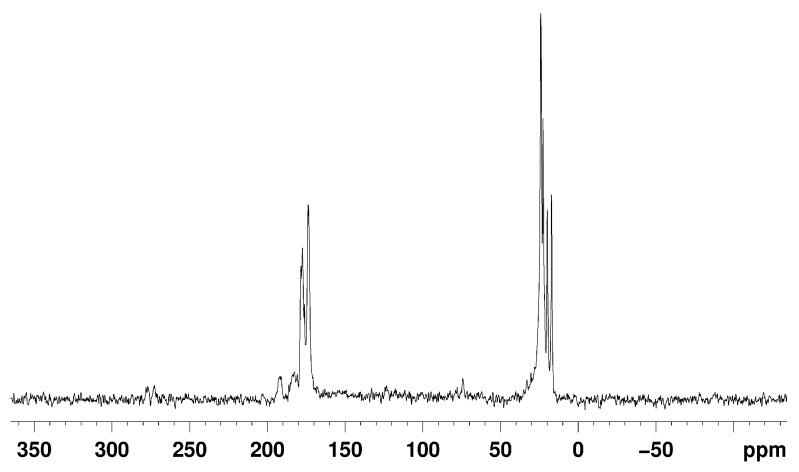
CP/MAS ^1^H-^13^C spectrum of AsPt blue.

Since the ^1^H NMR spectra, as mentioned above, were poorly resolved due to the excessive line broadening caused by oligomerization and thus scarcely informative, we decided to study the AsPt blue species by ^13^C and ^195^Pt NMR spectroscopy. Solid-state ^13^C and ^195^Pt NMR measurements were carried out on the Pt blue species and compared to those of AP-1. Despite several attempts, we were not able to obtain either solid-state or solution-state ^195^Pt NMR spectra of Pt blue. The investigated spectral window spanned from +2000 to −6000 ppm. The CP/MAS ^1^H-^13^C spectrum of Pt blue is shown in Figure 4 and consists of several peaks centered around 23 ppm (CH_3_) and 180 ppm (C(O)N), suggesting the presence of different species containing the acetamidate ligand. The existence of several species may account for the lack of detection of ^195^Pt NMR signals due to the intrinsic low sensitivity of the ^195^Pt nuclei. On the other hand, the good quality of the CP/MAS ^1^H-^13^C spectrum of Pt blue and the narrow linewidths indicate that the AsPt blue species is essentially diamagnetic, in agreement with the above observations. It is likely that the AsPt blue species possesses a complex architecture where the various acetamidate moieties may experience different kinds of local environments.

### 2.5. AsPt Blue: XPS Measurements

**Figure 5:**
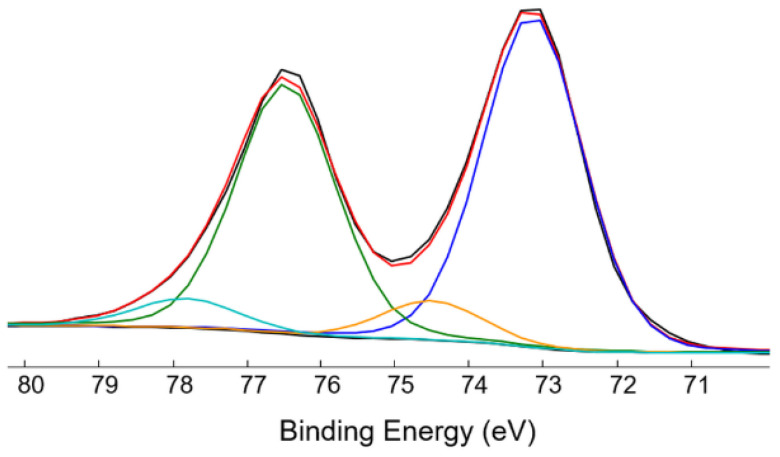
XPS spectrum and relevant curve fitting of Pt4f region recorded for AsPt blue.

The fact that the AsPt blue species is most likely an oxidation product of AP-1, the disappearance of the ^195^Pt NMR signals, and the evident diamagnetism suggest that this species might contain pairs of magnetically coupled Pt(III) centers in line with previous cases described in the literature [15,16,17]. This idea prompted us to exploit XPS spectroscopy to further analyze the Pt blue species and gain insight into the Pt oxidation state. The XPS spectrum of the Pt4f region recorded from a Pt blue sample is shown in Figure 5 and reveals that AsPt blue contains a mixture of high-oxidation-state Pt species. In particular, there is a main component with a doublet at BE(Pt4f_7/2_) = 73.2 ± 0.2 eV and BE(Pt4f_5/2_) = 76.5 ± 0.2 eV and a small contribution attributable to Pt(IV) with a doublet at BE(Pt4f_7/2_) = 74.5 eV ± 0.2 eV and BE(Pt4f_5/2_) = 77.8 ± 0.2 eV.

**Figure 6:**
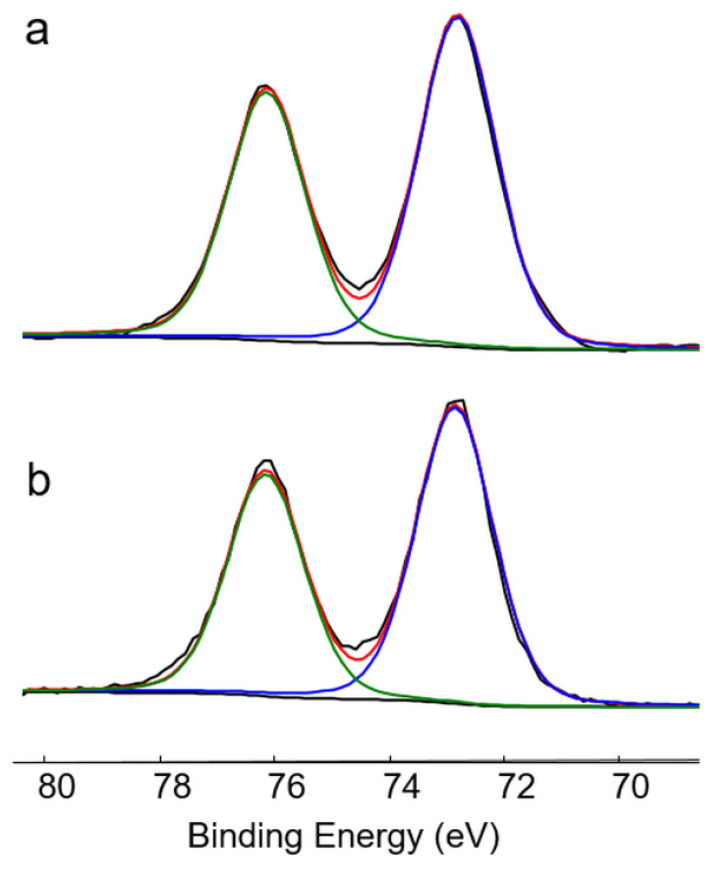
XPS spectra and relevant curve fittings of Pt4f regions recorded for Pt white (**a**) and AP-1 (**b**).

**Figure 7:**
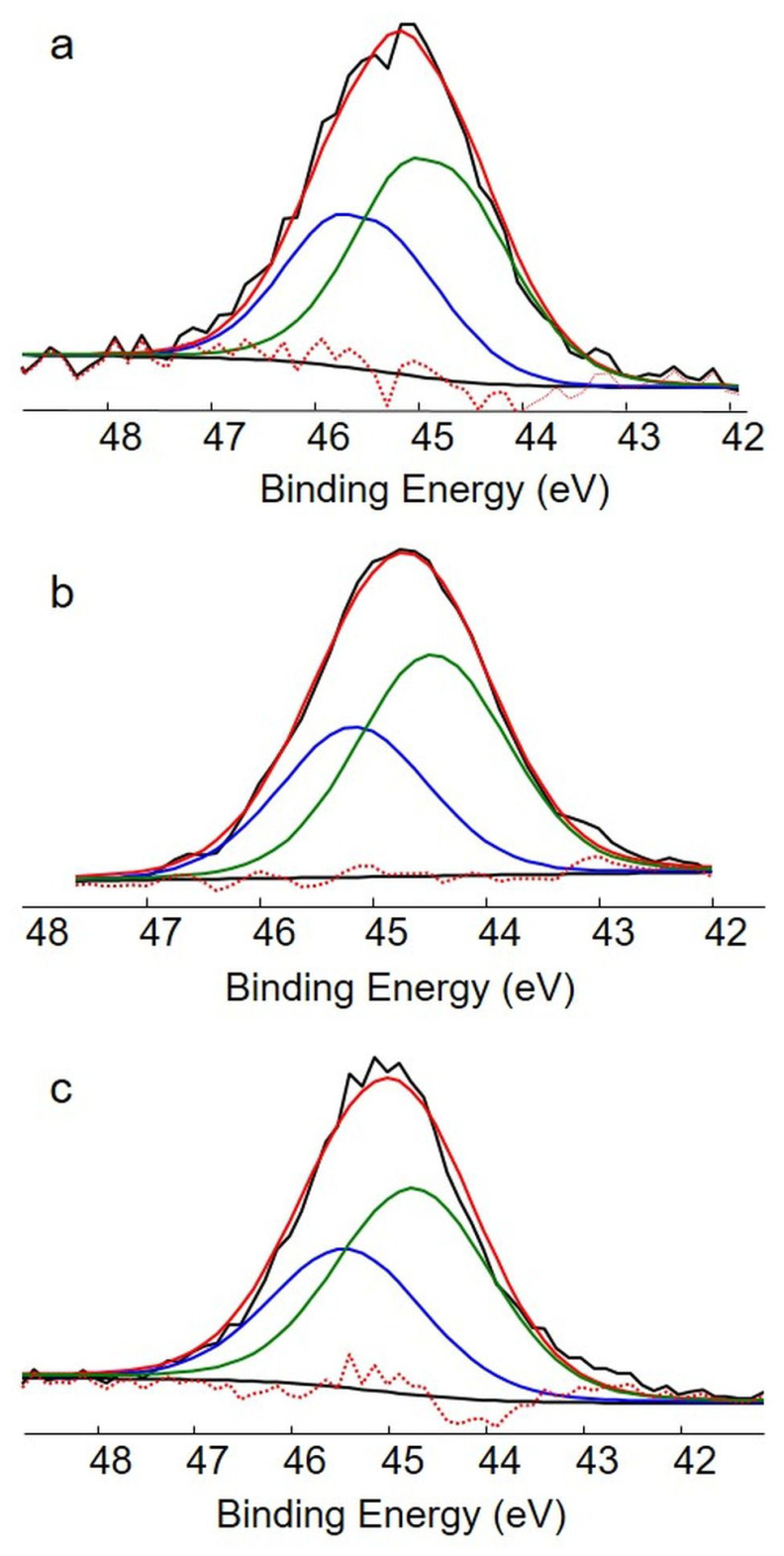
XPS spectra and relevant curve fittings of As3d regions recorded for Pt blue (**a**), Pt white (**b**), and AP-1 (**c**).

As far as the main component at 73.2 eV is concerned, this BE value is intermediate between those typical for Pt(II) and those for Pt(IV) species. This contribution can be attributed to a Pt(III) species, in agreement with what was observed by Stadnichenko et al. [18], and is approximately 90% of the total platinum amount. For comparison, the binding energies of the peaks of the doublet relevant to Pt white and AP-1 were found at 72.5 ± 0.2 eV and 75.8 ± 0.2 eV for Pt4f_7/2_ and Pt4f_5/2_ (Figure 6) and were attributed to the Pt(II) oxidation state [19]. In Figure 7, the XPS spectra of the As3d regions relevant to the Pt blue sample (a), Pt white (b), and AP-1 (c) are reported. Each signal was fitted with a doublet whose components, As3d_5/2_ and As3d_3/2_, showed a typical distance of 0.7 eV. The binding energies of the As3d_5/2_ peaks are very similar, i.e., 44.8 eV for Pt blue, 44.6 eV for Pt white, and 44.7 eV for AP-1, suggesting a formal arsenicum oxidation state of (III) in all the samples [20]. Moreover, considering the surface atomic percentages of platinum, arsenicum, and chlorine, these were recorded in all the investigated samples at a ratio of 1:1:1, as expected.

### 2.6. Pt Blue: Vibrational Spectroscopy

**Figure 8:**
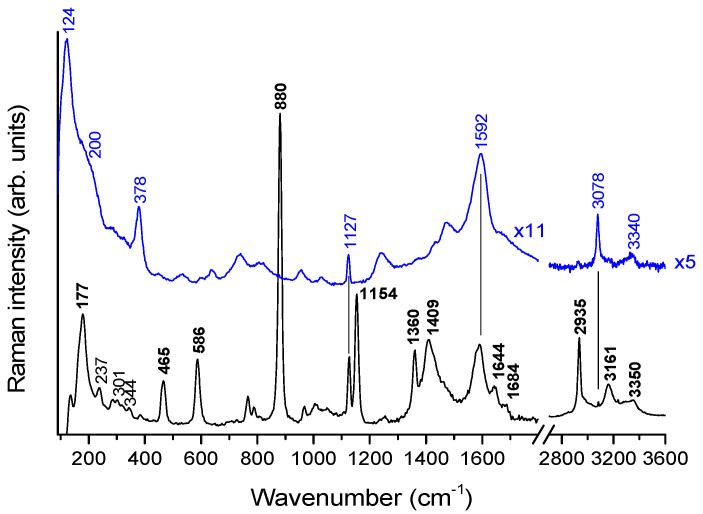
Raman spectra of AP-1 and AsPt blue solid sample (**upper** trace). Excitation wavelength = 514.5 nm, laser power = 300 μW, objective = 20×, acquisition time = 30 s, 5 accumulations.

The Raman spectra of AP-1 and AsPt blue were obtained in the solid phase upon excitation at 514.5 nm and are shown in Figure 8. The most intense bands observed in AP-1 and highlighted in bold in the figure are due to the vibrational modes of the acetamide molecules that crystallize in the unit cell. The signals at 1127, 1592, and 3078 cm^−1^ are assigned to the OCNH acetamidate moiety of AP-1. These bands are also observed in the Pt blue sample at the same wavenumbers, suggesting that the acetamidate moiety is not significantly perturbed by the oligomerization process. Indeed, in the CH stretching spectral region, the low-intensity band of the CH_3_ stretching vibrations can be related to the stiffening of the three-dimensional structure in the Pt blue species. The low-frequency spectral region of AP-1, between 230 and 400 cm^−1^, is characterized by vibrational modes mainly involving the platinum atom. These vibrations consist of the coupling of stretching Pt-Cl, Pt-As, or Pt-N with bending O-As-O [21,22]. In comparison with AP-1, the Pt blue compound shows a very strong band centered at 124 cm^−1^ with a shoulder around 200 cm^−1^.

According to previous data obtained for various Pt complexes [23,24,25,26], Raman frequencies in the range of 100–230 cm^−1^ can be considered specific signatures for the presence of Pt-Pt bonds with Pt in different oxidation states.

## 3. Materials and Methods

### 3.1. UV-Vis Experiments

The solution behavior of AP-1 was assessed through spectrophotometric studies performed with a Cary 50 Bio UV-Vis spectrophotometer (Varian, Palo Alto, CA, USA) in the presence of 10 mM phosphate buffer, pH = 7.4. The obtained solution of AP-1 (5 × 10^−4^ M) was monitored in the wavelength range between 200 and 800 nm for 72 h at 25 °C.

### 3.2. NMR Experiments

Solid-state NMR analyses were performed on a Avance I 400 spectrometer (Bruker Biospin GMBH, Rheinstetten, Germany) (operating at a frequency of 100.6 MHz for ^13^C and 86.0 MHz for ^195^Pt) using a 4.0 mm HX MAS probe at 298 K. For the MAS and static (non-spinning) experiments, the samples were packed into zirconia rotors.

The chemical shifts for ^13^C were referenced against SiMe_4_ (0 ppm) by using the methylene signal of adamantane (δ 38.48) as a secondary reference, while the ^195^Pt chemical shifts were referenced against H_2_PtCl_6_.

The ^1^H-^13^C CP/MAS NMR experiments were acquired using a 3.25 μs proton *π*/2 pulse length, an ν_CP_ of 55.0 kHz, a contact time of 5.0 ms, an ν_dec_ of 76.9 kHz, and a recycle delay of 6.0 s. The spinning rate for the ^1^H-^13^C CP/MAS NMR spectra was 10,000 Hz.

The static solid-state ^195^Pt NMR experiments were performed using the Cross-Polarization Carr–Purcell–Meiboom–Gill (CP/CPMG) pulse sequence [27,28]. The ^1^H-^195^Pt CP/CPMG spectra were obtained by collecting subspectra with a spectral width of 75 kHz and 50 Meiboom–Gill (MG) loops. For Pt white, seventeen subspectra were acquired using transmitter offsets spaced by 30 kHz (the first transmitter offset was set at −81,714.33 Hz). The subspectra were co-added using the skyline projection method. The acquisition time (1/τ_a_) was adjusted to attain a spikelet separation of 4.4 kHz. The ^195^Pt spectra were obtained using a 3.25 µs proton π/2 pulse length, a ν_CP_ of 65.4 kHz, a contact time of 7.0 ms, an ν_dec_ of 77.0 kHz, and a recycle delay of 6 s. A two-pulse phase modulation (TPPM) decoupling scheme was used for the ^1^ H decoupling.

### 3.3. XPS Experiments

XPS analyses were performed on a scanning microprobe PHI 5000 VersaProbe II (Physical Electronics, Chanhassen, MN, USA). The instrument was equipped with a micro-focused monochromatized AlKα X-ray radiation source. The samples were examined in HP mode with an X-ray take-off angle of 45° (instrument base pressure = ~10^−9^ mbar). The size of the scanned area was about 1400 µm × 200 µm. Wide scans and high-resolution spectra were recorded in FAT mode for each sample, setting the pass energy values equal to 117.4 eV and 29.35 eV, respectively. To fit the high-resolution spectra, the commercial MultiPak software, version 9.9.0.8, was used. Atomic percentages were inferred from the peak areas, previously normalized by MultiPak library’s sensitivity factors. Adventitious carbon C1s was set at 284.8 eV and used as a reference.

### 3.4. ICP-OES Measurements for Pt and As

The determination of the Pt and As concentrations was performed using a Varian 720-ES inductively coupled plasma atomic emission spectrometer (ICP-OES) (Varian, Palo Alto, CA, USA) equipped with a CETAC U5000 AT+ ultrasonic nebulizer (Teledyne, Omaha, NE, USA) in order to increase the method’s sensitivity.

A total of 100 µL of each of the aqueous solutions containing the Pt blue and Pt white species was transferred into PE vials and digested in a thermo-reactor at 80 °C for 8 h with 2 mL of aqua regia (HCl supra-pure grade and HNO_3_ supra-pure grade at a 3:1 ratio). After mineralization, ultrapure water (≥18 MΩ) was added to a final volume of 6 mL. All the samples were spiked with 1 ppm of Ge, used as an internal standard, and analyzed. Calibration standards were prepared through gravimetric serial dilution from a commercial standard solution of Pt and As at 1000 mg L^−1^. The following wavelengths were used: 214.424 nm for Pt, 188.980 nm for As, and 209.426 nm for Ge. The operating conditions were optimized to obtain the maximum signal intensity, and between each sample, a rinsed solution of HCl supra-pure grade and HNO_3_ supra-pure grade at a 3:1 ratio was used to avoid any “memory effect”.

### 3.5. Raman Measurements

Raman measurements were performed by means of a Renishaw 2000 spectrometer (Renishaw plc, Wotton-under-Edge, UK) equipped with the 514.5 nm line from an argon laser and an incident power of 300 μW, coupled with a Leica DLML confocal microscope (Leica, Wetzlar, Germany) with a 20× objective. The back-scattered Raman signal was collected and focused into a single grating monochromator (1200 lines mm^−1^) through 40 μm slits and detected using a Peltier-cooled CCD detector at −20 °C. The spectrometer was routinely calibrated with respect to the 520 cm^−1^ band of a silicon wafer.

### 3.6. EPR Experiments

The EPR spectra at X-band (ca. 9.4 GHz) were acquired using an Elexsys E500 spectrometer (Bruker Gmbh, Billerica, MA, USA) equipped with an SHQ cavity and an ESR900 continuous-flow cryostat for low-temperature operation. The sample was prepared by transferring the AsPt blue solution into a standard EPR tube. Measurements were conducted both at room temperature and at 30 K, freezing the sample in liquid nitrogen before its insertion into the cavity.

## 4. Conclusions

On the basis of the data collected so far, the following interpretation of the process of AsPt blue formation can be proposed. In aerobic aqueous solutions, AP-1 may undergo a slow but progressive conversion into the so-called AsPt blue species. The latter appears to be the result of combined oligomerization and oxidation processes. Interestingly, an oligomeric white species is obtained in the absence of dioxygen, which was previously characterized. Based on the results reported here, we can confirm that AsPt blue is an oligomeric species with a MW > 10 kDa. This species is mostly diamagnetic and is characterized by an intense absorption band at 600 nm. Analytical determinations revealed significant losses of platinum and arsenic in AsPt blue compared to AP-1. Despite the presence of some apparent heterogeneity, most likely due to the concomitant oligomerization/oxidation processes, we propose that the platinum centers in AsPt blue are predominantly in the +3 oxidation state, probably as diamagnetic Pt(III) pairs. This hypothesis is broadly supported by the XPS results and the ^13^C NMR spectra. The vibrational spectra provide further support for the Pt-Pt bond formation hypothesis by revealing a low Raman shift band specific to the Pt-Pt interaction. Attempts are being made to further characterize this species from a structural point of view. In any case, we have gathered enough evidence to document that this species is non-canonical Pt blue, essentially diamagnetic in nature.

## Figures and Tables

**Table 1:** Elemental composition of the studied Pt-As compounds.

	C	H	N	O	Cl	Pt	As
**AsPt blue**	10.67	2.49	5.15	25.00	7.01	36.70	10.80
**Pt white**	11.09	2.38	5.94	22.21	-	43.66	17.43
AP-1 *	10.55	2.21	6.15	14.05	7.78	42.82	16.44

* without acetamide.

## Data Availability

The data are contained within the article and Supplementary Materials.

## Supplementary Materials

The supporting information can be downloaded at https://www.mdpi.com/article/10.3390/ijms25137408/s1.
