# Supplementary material for: Description of a Non-Canonical AsPt Blue Species Originating from the Aerobic Oxidation of AP-1 in Aqueous Solution
Source: Int J Mol Sci. 2024 Jul 5;25(13):7408. doi: 10.3390/ijms25137408 (PMC11242394; doi:10.3390/ijms25137408)
Supplement: Supplementary file 1 [file ijms-25-07408-s001.zip › ijms-3048273-supplementary.pdf]

Supporting Information

Description of a non-canonical AsPt blue species originating from aerobic oxidation of AP-1 in aqueous solution

Damiano Cirri, Tiziano Marzo, Piero Mastrorilli, Valentina Petrelli, Stefano Todisco, Elvira De Giglio, Cristina Gellini, Marilena Ricci, Alessandro Pratesi and Luigi Messori

Content:

UV-Vis experiments – fitting parameters 2

Filtration test 2

UV-Vis experiments – fitting parameters

Model	Logistic		
Equation	y = A2 + (A1-A2)/(1 + (x/x0)^p)		
Reduced Chi-Sqr	4,48917E-5		
Adj. R-Square	0,99377		
		Value	Standard Error
A	A1	0,00116	0,00369
A	A2	0,27985	0,02099
A	x0	12,58092	0,52381
A	p	4,18844	0,45058
A	EC20	9,03585	
A	EC50	12,58092	
A	EC80	17,51684	

Table. S1 Fitting parameters used for the inset in Figure 2.

Filtration test

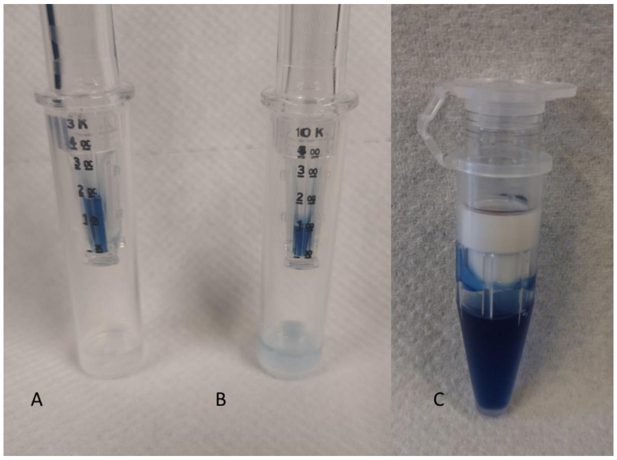

Figure S1. Filtration tests on filters with cutoffs of 3 kDa (**A**), 10 kDa (**B**) and 100 KDa (**C**).
